# Crenigacestat blocking notch pathway reduces liver fibrosis in the surrounding ecosystem of intrahepatic CCA viaTGF-β inhibition

**DOI:** 10.1186/s13046-022-02536-6

**Published:** 2022-11-28

**Authors:** Serena Mancarella, Isabella Gigante, Grazia Serino, Elena Pizzuto, Francesco Dituri, Maria F. Valentini, Jingxiao Wang, Xin Chen, Raffaele Armentano, Diego F. Calvisi, Gianluigi Giannelli

**Affiliations:** 1 National Institute of Gastroenterology “S. De Bellis” Research Hospital, Via Turi 27, 70013 Castellana Grotte, BA Italy; 2 Department of Emergency and Organ Transplant, University of Bari Medical School, Bari, Italy; 3 Department of Bioengineering and Therapeutic Sciences and Liver Center, University of California, San Francisco, CA 94143 USA; 4 Institute of Pathology, University of Regensburg, 93053 Regensburg, Germany

**Keywords:** Tissue microenvironment, Liver fibrosis, Tumor stroma crosstalk, Crenigacestat, Smad2

## Abstract

**Background:**

Intrahepatic cholangiocarcinoma (iCCA) is a highly malignant tumor characterized by an intensive desmoplastic reaction due to the exaggerated presence of the extracellular (ECM) matrix components. Liver fibroblasts close to the tumor, activated by transforming growth factor (TGF)-β1 and expressing high levels of α-smooth muscle actin (α-SMA), become cancer-associated fibroblasts (CAFs). CAFs are deputed to produce and secrete ECM components and crosstalk with cancer cells favoring tumor progression and resistance to therapy. Overexpression of Notch signaling is implicated in CCA development and growth. The study aimed to determine the effectiveness of the Notch inhibitor, Crenigacestat, on the surrounding microenvironment of iCCA.

**Methods:**

We investigated Crenigacestat’s effectiveness in a PDX model of iCCA and human primary culture of CAFs isolated from patients with iCCA.

**Results:**

In silico analysis of transcriptomic profiling from PDX iCCA tissues treated with Crenigacestat highlighted “liver fibrosis” as one of the most modulated pathways. In the iCCA PDX model, Crenigacestat treatment significantly (*p* < 0.001) reduced peritumoral liver fibrosis. Similar results were obtained in a hydrodynamic model of iCCA. Bioinformatic prediction of the upstream regulators related to liver fibrosis in the iCCA PDX treated with Crenigacestat revealed the involvement of the TGF-β1 pathway as a master regulator gene showing a robust connection between TGF-β1 and Notch pathways. Consistently, drug treatment significantly (*p* < 0.05) reduced TGF-β1 mRNA and protein levels in tumoral tissue. In PDX tissues, Crenigacestat remarkably inhibited TGF-β signaling and extracellular matrix protein gene expression and reduced α-SMA expression. Furthermore, Crenigacestat synergistically increased Gemcitabine effectiveness in the iCCA PDX model. In 31 iCCA patients, TGF-β1 and α-SMA were upregulated in the tumoral compared with peritumoral tissues. In freshly isolated CAFs from patients with iCCA, Crenigacestat significantly (*p* < 0.001) inhibited Notch signaling, TGF-β1 secretion, and Smad-2 activation. Consequently, Crenigacestat also inactivated CAFs reducing (*p* < 0.001) α-SMA expression. Finally, CAFs treated with Crenigacestat produced less (*p* < 005) ECM components such as fibronectin, collagen 1A1, and collagen 1A2.

**Conclusions:**

Notch signaling inhibition reduces the peritumoral desmoplastic reaction in iCCA, blocking the TGF-β1 canonical pathway.

**Supplementary Information:**

The online version contains supplementary material available at 10.1186/s13046-022-02536-6.

## Background

Intrahepatic cholangiocarcinoma (iCCA) is a highly lethal tumor that originates from the internal bile ducts of the liver. Although still considered a rare tumor entity, over the past 20 years, the iCCA incidence and mortality rates have considerably increased in most locations worldwide [1], likely because of the association with HBV- and/or HCV-related liver diseases and, more recently, with metabolic liver disorders [2, 3]. The rate of increased iCCA occurrence in Italy is the highest in Europe [4, 5]. iCCAs typically exhibit a massive desmoplastic reaction characterized by an abundant deposition of extracellular matrix (ECM) components, including collagen (COL1A1, COL1A2) and fibronectin (FN), by cancer-associated fibroblasts (CAFs), a critical component of the tumor microenvironment. iCCA CAFs are specialized myofibroblasts with high levels of alpha-smooth muscle actin (α-SMA), which presumably derive from activated Hepatic Stellate Cells (HSCs) and portal and periductal liver fibroblasts [6–11]. CAFs crosstalk with tumor cells through cytokines, chemokines, growth factors, and extracellular vesicles that promote tumor progression and epithelial-mesenchymal transition (EMT) of cancer cells [12], leading to therapy resistance [7, 8, 13, 14]. Therefore, high α-SMA expression in iCCA human tissues is correlated with the worst prognosis and poorer survival outcomes in iCCA patients following surgical resection [15].

Transforming growth factor (TGF)-β1 signaling is the prominent driver of fibrogenesis in various organs, such as the liver, kidney, and lungs [16–18]. At the molecular level, TGF-β1 exerts its biological effects by binding to transforming growth factor β receptor II (TGFβRII), which phosphorylates transforming growth factor β receptor I (TGFβRI). The latter, in turn, activates cytoplasmic Smad2 and/or Smad3 proteins, which form a heterotrimeric complex with Smad4 and translocate to the nucleus to regulate gene transcription [16]. In the liver, TGF-β1 is a well-characterized profibrogenic cytokine converting fibroblasts into myofibroblasts [19–24] and orchestrating tissue homeostasis by producing and depositing ECM components.

In the liver, Notch signaling is critical for the proper development of the biliary tree. In addition, recent studies in preclinical experimental models showed that Notch signaling dysregulation is implicated in liver regeneration and repair, liver fibrosis, and CCA development [25–27]. However, clinical data reporting the therapeutic effectiveness of targeting such a pathway in patients with CCA are lacking [28–30]. Activation of the Notch pathway leads to the proteolytic cleavage of the Notch intracellular domain (NICD) and its translocation into the nucleus, promoting the transcription of target genes as the helix-loop-helix transcription factor HES-1 [31]. Furthermore, Notch regulates juxtacrine and paracrine communications between tumor cells and tumor stroma. The overexpressed Notch pathway orchestrates the activation of other signals and different cell types that surround the tumor mass, such as the CAFs, which are responsible for the reactive stroma [31]. This scenario suggests that targeting CAFs activation and/or recruitment at iCCA tumor sites may offer new therapeutic strategies and potential therapeutic benefits for controlling iCCA aggressiveness caused by activated CAFs. Crenigacestat, a selective Notch1-γ-secretase inhibitor (GSI), has been tested in a phase 1 clinical trial in patients with advanced or metastatic solid tumors, including CCA (NCT02784795, https://clinicaltrials.gov/ct2/show/NCT02784795). Recently, we have reported that Crenigacestat inhibits the growth of iCCA in PDX and xenograft models by modulating angiogenesis and/or the expression of stemness marker CD90 [32, 33]. No data are currently available regarding the role of CAFs crosstalk with epithelial iCCA cells, although iCCA lesions are characterized by a robust desmoplastic reaction. The study investigates the effectiveness of inhibiting the Notch pathway on the ecosystem surrounding iCCA.

## Methods

### Human iCCA tissues

This study falls under the approval of the local ethics committee, Azienda Ospedaliero Universitaria Consorziale Policlinico di Bari (Bari, Italy); protocol number: 254; date of release: February 2012. Immediately after surgical resection, iCCA tissue specimens were cut into 0.5–1 cm pieces and stored in MACS tissue storage solution (Miltenyi Biotec, Bergisch Gladbach, Germany). These tissue fragments were cut into smaller pieces (1–2 mm). Then, the iCCA tissue pieces were directly implanted in mice or processed to enhance the number of cancer-associated fibroblasts.

### Establishment of the patient-derived xenograft (PDX) model

Primary tumor explanted from a patient was collected and transferred to Hank’s Balanced Salt Solution, then cut using a sterile scalpel in pieces smaller than 1 cm, and collected in cryovials at − 80 °C and after a few days in liquid nitrogen. The development of the PDX model, after approval of the Ethical Committee (Prot. N. 254/C.E), was conducted at the Biogem Animal House in Ariano Irpino (Avellino, Italy), under the National Academy of Sciences guidelines. Tissue fragments were implanted subcutaneously in the flanks of 4–5-week-old CD1 immunodeficient nude female mice. During the study, each mouse was given drinking water ad libitum and a complete pellet diet (GLP 4RF21, Mucedola). Mice were monitored daily for clinical signs and mortality, and body weight (BW) was assessed weekly. Tumor growth was controlled every 2 weeks with Mitutoyo forceps. Experiments ended 8 weeks after the tumor implant, sacrificing animals with tumor masses greater than 15% of BW and/or with a body weight loss (BWL) of 10%. All animals were weighed every 2–3 days during the experimental period. The BWL was determined as follows: body weight loss percent (% BWL max) = 100 − (mean BW day x/mean BW day 1 × 100), where BWx is the mean BW at the day of maximal loss during the experiment, and BW1 is the mean BW on the first day of the experimental period. At the end of the study, mice were sacrificed by cervical dislocation, and the tumor masses were photographed and collected. Tumor tissues of 100 mm3 were implanted, and after engraftment, the mice were divided into two groups of ten animals each and treated with vehicle only or Crenigacestat (LY3039478, Selleckchem Chemicals, Houston, TX, USA) (8 mg/kg) or Gemcitabine (Selleckchem Chemicals, Houston, TX, USA) (125 mg/kg) or the combination of the two drugs together. The Tumor Volume (TV) was calculated using the formula: TV (mm3) = [length (mm) × width (mm)2]/2, where width and length are the shortest and longest diameters.

### Hydrodynamic model of iCCA

Hydrodynamic iCCA model was generated as described previously [25, 34]. FVB/N mice were purchased from the Jackson Laboratory. Primary iCCA was induced using hydrodynamic tail vein injection with the combination of AKT (10μg), Jagged1 (40μg), and SB (2μg) plasmids. Mice were given the Notch inhibitor (Crenigacestat/LY3039478) 8 mg/kg or vehicle orally on week 9 every two days for 3 weeks. All mice were sacrificed on week 12 or when moribund. Body weight and liver weight were recorded. Tumor tissues were preserved for further analysis. Mice were maintained and monitored following protocols approved by the Committee for Animal Research at the University of California, San Francisco (San Francisco, CA).

### hCAF isolation

CAF isolation from iCCA tissue was performed as previously described [35]. iCCA tissue specimens were subjected to enzymatic and mechanical digestion in HBSS solution with 50–200 U/mL collagenase Type IV (Thermo Fisher Scientific, Waltham, MA, USA), 3 mM CaCl2, and Antibiotic–Antimycotic (Thermo Fisher Scientific, Milan, Italy) at 37 °C under gentle rotation for 2 h or more as needed. The resulting cells were harvested and washed three times with HBSS by centrifugation, resuspended in IMDM with 20% FBS, and kept on ice. At the end of this step, the fibroblasts in the supernatant were centrifuged at 500×g for 10 min. Recovered CAFs were then cultured in complete minimum essential medium (IMDM), a modified Dulbecco’s modified Eagle medium (DMEM) with 20% fetal bovine serum (FBS, Thermo Fisher Scientific, Waltham, MA, USA) and Antibiotic–Antimycotic. CAFs isolated from multiple patients were treated in a serum-free medium with vehicle or different concentrations (1–5-10 μM) of Crenigacestat, or various concentrations (5–10-20 μM) of FLI-06. Conditioned media produced by these cells were also collected and concentrated using centrifugal filter devices (Amicon ultra-15 centrifugal filters ultracel-3 K, MerckMillipore, Burlington, Massachusetts, USA).

### Masson’s trichrome and immunofluorescence staining

To analyze the grade of tissue fibrosis in PDX iCCA tissues, vehicle and Crenigacestat treated Masson’s trichrome staining was performed with Mallory trichrome acc. McFarlane kit (DIAPATH) following the manufacturer’s instructions. The degree of fibrosis was classified as mild, moderate, or severe. The images were acquired with the Eclipse Ti2 microscope (Nikon Inc., Melville, NY, USA) using an × 20 objective lens.

Immunofluorescence on iCCA PDX tissues was performed as previously described [36]. Tissues were fixed in a 1:1 acetone:chloroform solution, blocked with 2% bovine serum albumin solution. The slides were stained with α-SMA (1:200, Sigma, St. Louis, Missouri, USA), Vimentin antibodies (1:100, Cell Signaling Technologies, Massachusetts, USA), TGF-β1 (1:1000, Thermo Fisher Scientific, Waltham, MA, USA) and pSMAD2 (1:1000, Abcam, Cambridge, UK). For all stainings, the percentage of positively stained cells in treated slides was normalized on the positive signal in untreated slides.

α-SMA and Vimentin protein expression was also analyzed in iCCA hCAFs seeded in chamber slides by immunofluorescence staining as previously described [33]. Cells were fixed with PFA4% and permeabilized with 0.1% Triton X-100 in PBS in 2% bovine serum albumin for 30 min. Afterward, CAFs slides were incubated with α-SMA (1:200, Sigma, St. Louis, MO, USA) and Vimentin antibodies (1:100, Cell Signaling Technologies, Danvers, MA, USA). After washing, both iCCA PDX tissues and CAFs were incubated with the appropriate secondary immunoglobulin G H&L (Alexa Fluor 488, Thermo Fisher Scientific, Waltham, MA, USA). Nuclei were stained with 4′,6-diamidino-2-phenylindole (DAPI)-supplemented antifade mounting medium VECTASHIELD (Vector Lab, Burlingame, CA, USA).

The Eclipse Ti2 microscope (Nikon Inc., Melville, NY) was used to visualize histological samples. Five images were captured in different positions for each sample, and staining was quantified using ImageJ analysis software.

### RNA extraction

Total RNA was extracted using TRIzol® (Thermo Fisher Scientific) according to the manufacturer’s instructions. The RNA concentration was determined with the NanoDrop Spectrophotometer (Thermo Fisher Scientific).

### Quantitative reverse-transcription real-time PCR (qRT-PCR)

cDNA was obtained starting from 2 μg of total RNA, using the High-Capacity cDNA Reverse Transcription kit (Applied Biosystems by Thermo Fisher Scientific) according to the manufacturer’s instructions. Quantitative PCR reactions were performed using the iTaq Universal SYBR Green Supermix (Biorad Laboratories, Hercules, CA, USA) and the primers for HES1 (Hs00172878_m1) (Applied Biosystems, Foster City, CA, USA), GAPDH (qHsaCED0038674), FN1 (qHsaCED0043611), COL1A1 (qHsaCED0043248), and COL1A2 (qHsaCED0003988) (Biorad Laboratories, Hercules, CA, USA), and primer sequences for TGFB1: forward, 5′-GGAAATTGAGGGCTTTCGCC-3′; reverse, 5′-GGTAGTGAACCCGTTGATGTCC-3′. Experiments were repeated three times in triplicate. Relative expression was calculated using the 2 − ΔΔCt method.

### Protein extraction and Western blot analysis

Protein expression was studied on purified cell lysates and concentrated conditioned media. Cell total proteins were extracted using the T-PER Tissue Protein Extraction Reagent (ThermoFisher Scientific) with the Halt Protease & Phosphatase Inhibitor (ThermoFisher Scientific). Proteins were separated in 4–20% Tris-glycine sodium dodecyl sulfate-polyacrylamide gel (Bio-Rad Laboratories, Hercules, CA). Membranes were incubated with the following antibodies: human primary anti-Notch cleaved 1 (1:1000, Cell Signaling Technology, Pero, Italy); purified human anti-HES1 (1:1000, Cell Signaling Technology); anti-TGFβ (1:500 R&D); anti-phosphoSMAD2 (1:1000, Abcam, Cambridge, UK), anti-SMAD2/3 (1:1000, Cell Signaling Technology) and anti-glyceraldehyde-3-phosphate dehydrogenase (GAPDH) (1:1000, Santa Cruz Biotechnology, Santa Cruz, CA). In addition, a secondary anti-rabbit (1:2000, Cell Signaling Technology) or anti-mouse (1:2000, Biorad Laboratories, Hercules, CA, USA) antibody was used. The chemiluminescence signal from proteins was revealed using Clarity Max Western ECL Substrate (Bio-Rad) and captured with the ChemiDoc MP instrument (Bio-Rad Laboratories) using Image Lab 5.2.1. The relative density of the bands was calculated using the ImageLab software.

### Bioinformatic and statistical analysis

Ingenuity pathway analysis (IPA) software (Qiagen, USA) was used to identify the pathways and the upstream transcriptional regulators modulated by Crenigacestat.

Biological and technical replicates were analyzed with the most appropriate statistical tests (i.e., test-t or ANOVA) to establish statistical significance and reproducibility. A *p*-value ≤0.05 was considered statistically significant. The GraphPad Prism 5.0 software (La Jolla, CA, USA) was used to perform all statistical analyses.

## Results

### Crenigacestat affects the peritumoral ecosystem in iCCA models

**Fig. 1:**
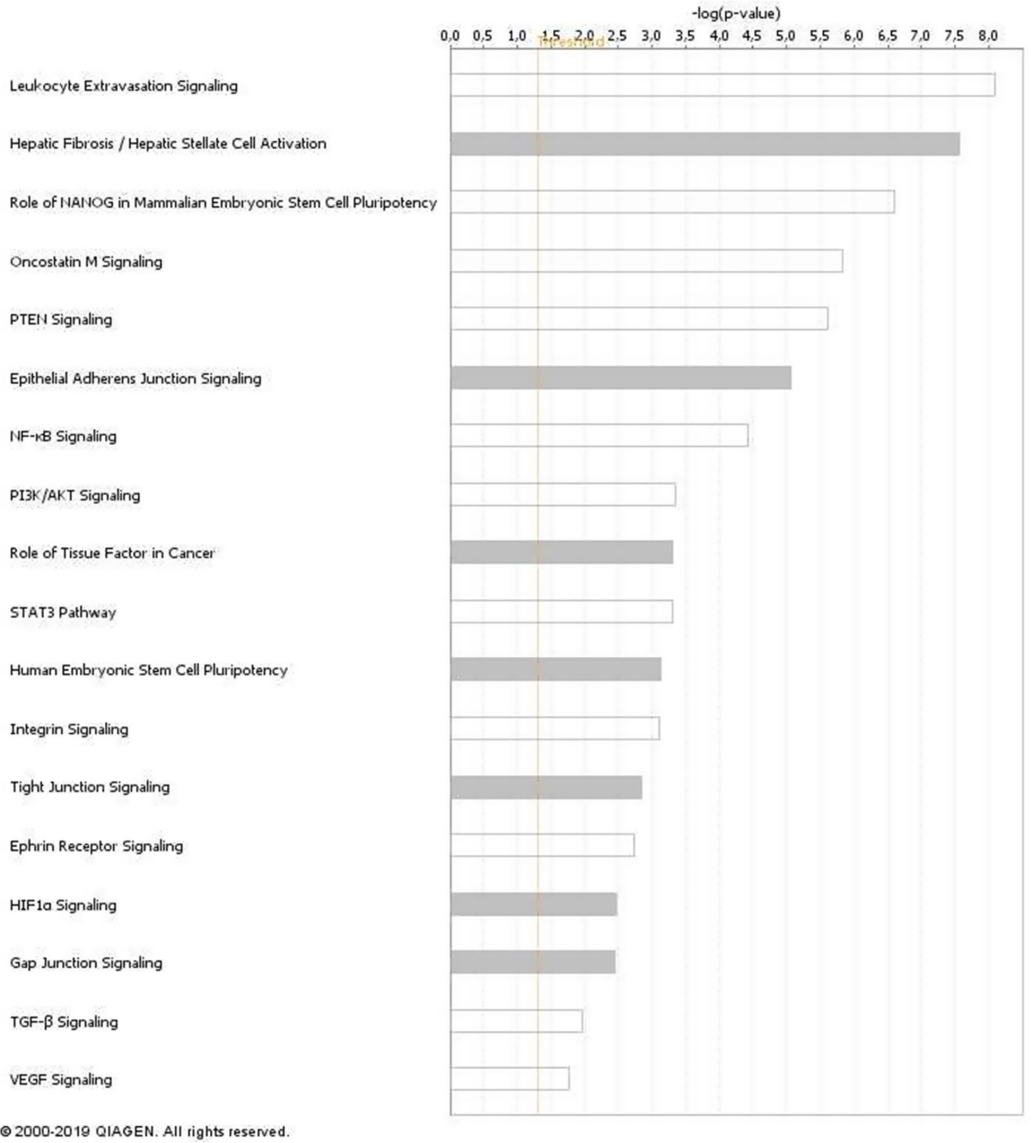
Canonical pathways analysis of the differentially expressed genes filtered based on their expression in liver and cancer. The most significantly enriched canonical pathways based on -log *p* values are displayed

**Fig. 2:**
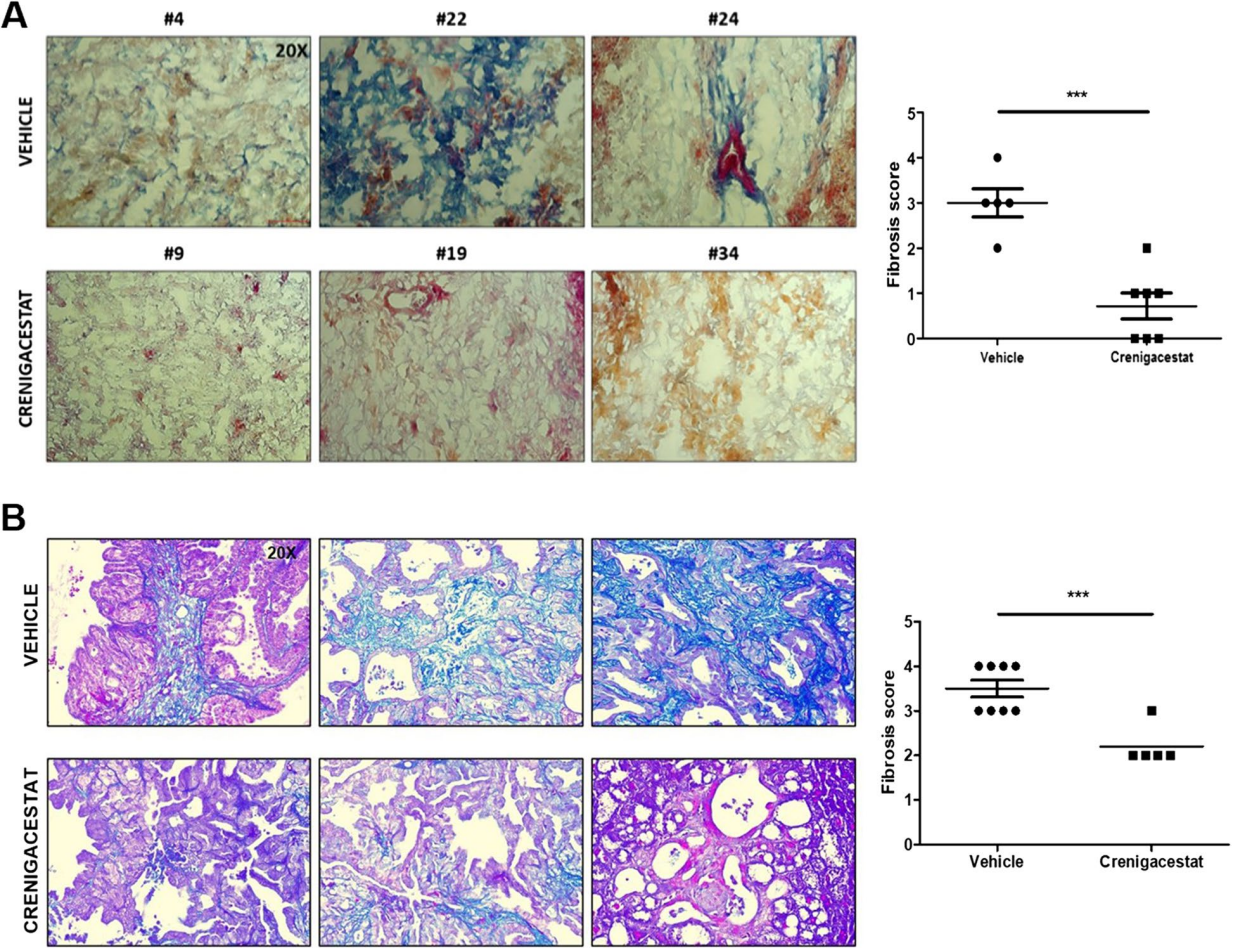
Crenigacestat reduced peritumoral fibrosis in two iCCA models. Panel **A**. PDX iCCA model and Panel **B**. Hydrodynamic injection iCCA model. Tissue sections were stained using the Azan Mallory’s trichrome staining. Representative images were acquired at 20X magnification. Scale bar represents 50 μm. An adapted METAVIR scoring system was used to quantify the fibrosis in treated and untreated mice, as reported in the graphs

Based on the transcriptomic profile we generated of iCCA tissues in PDX model after Crenigacestat treatment (GSE134114) [32, 33], we performed a pathway analysis on differentially expressed genes using the filters “LIVER” and “CANCER”. This analysis showed that one of the most significant canonical pathways modulated by Crenigacestat was “Hepatic Fibrosis/Hepatic Stellate Cell Activation” (Fig. 1). This finding prompted us to investigate the desmoplastic reaction in PDX models. Notably, Crenigacestat treatment reduced liver fibrosis as compared to vehicle-treated animals, and such a difference was also statistically significant (*p* < 0.0001) using METAVIR score to quantify liver fibrosis (Fig. 2A). Furthermore, in a hydrodynamic mouse model in which the co-transfection of myr-AKT and Notch ligand Jag1 in the mouse liver induces iCCA development, Crenigacestat treatment reduced the associated peritumoral fibrosis (Fig. 2B). In conclusion, Crenigacestat reduces the fibrotic tissue in the microenvironment surrounding iCCA in PDX and hydrodynamic models.

### Crenigacestat inhibits the TGF-β1 pathway and deactivates CAFs in an iCCA PDX model

**Fig. 3:**
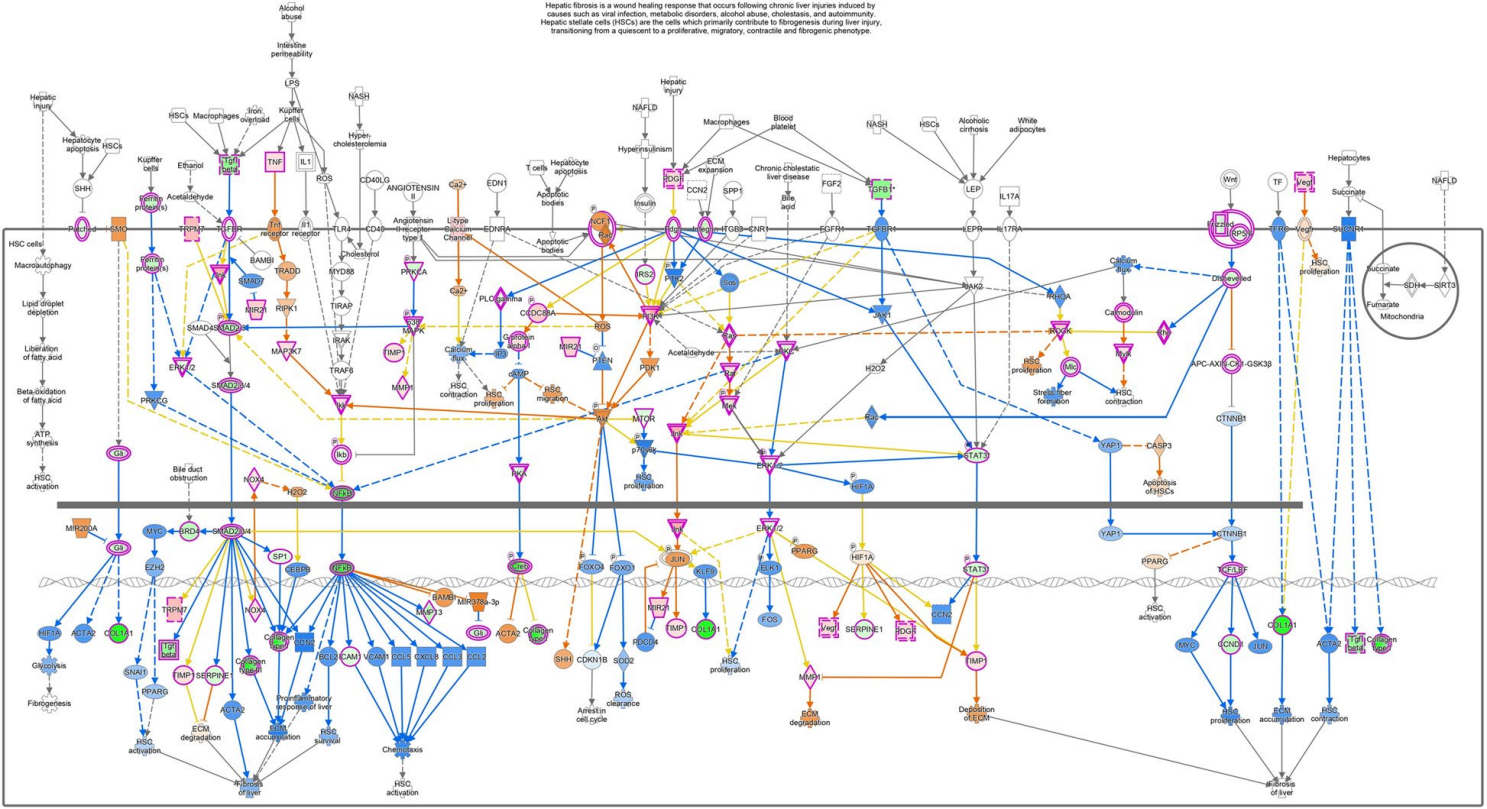
“Hepatic Fibrosis/Hepatic Stellate Cell Activation” was the most significant canonical pathways modulated by Crenigacestat in iCCA PDX mouse models

**Fig. 4:**
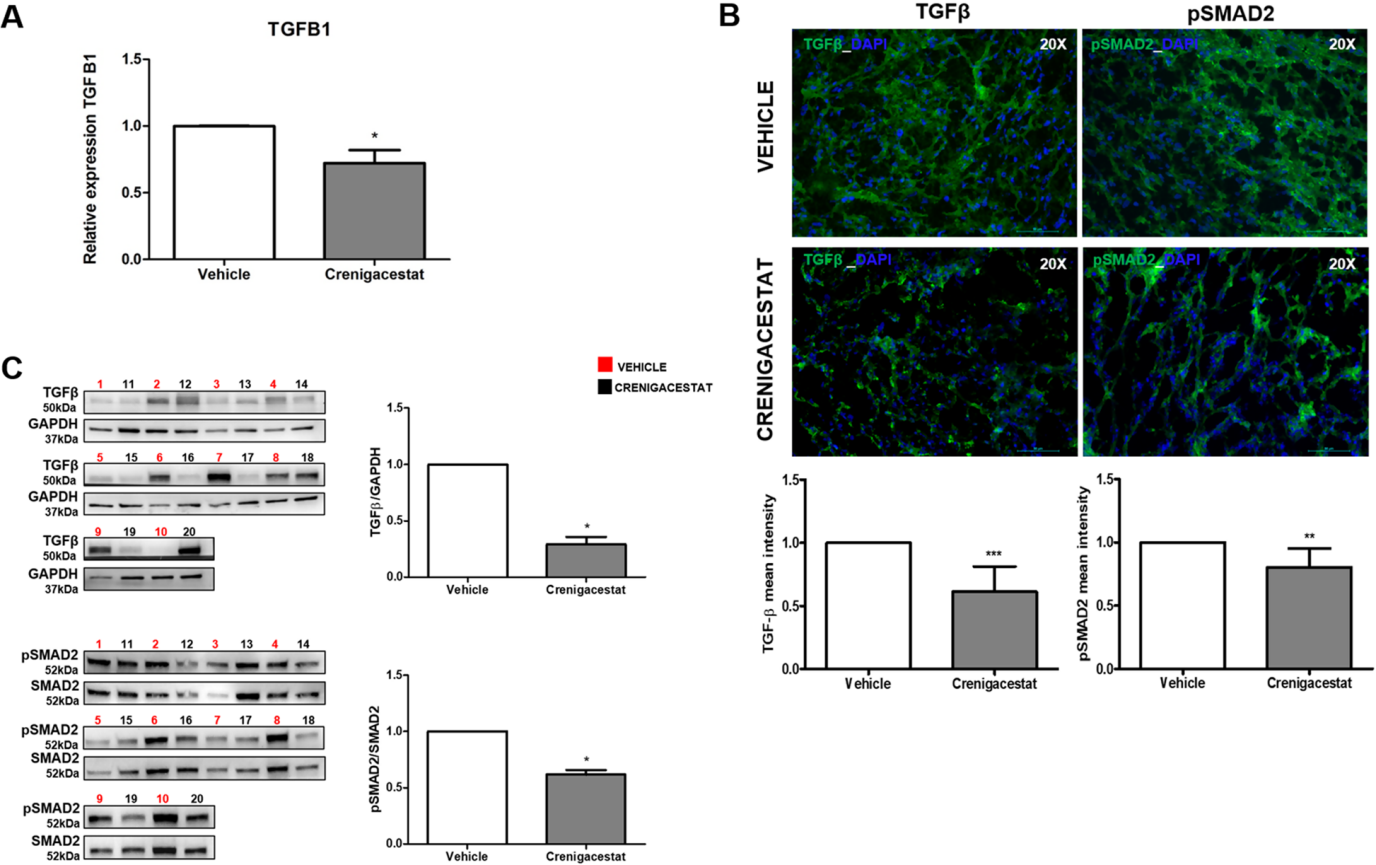
Crenigacestat inhibits the TGF-β pathway in iCCA PDX models. Panel **A**. Real-time PCR for TGFB1 on PDX mice reveals a reduction of TGF-β gene expression in PDX mice treated with Crenigacestat. Panel **B** and **C**. Crenigacestat reduces the TGF-β/Smad pathway in PDX-treated mice, as detected by Western blot analysis and immunofluorescence staining. The scale bar represents 50 μm. ***p < 0.0001. Magnification 20X

To explore the molecular mechanism responsible for the previously described results, we investigated the most relevant genes involved in the “HEPATIC FIBROSIS” pathway. As illustrated in Fig. 3, the most potent fibrogenic cytokine was TGF-β1, which was significantly downregulated after treatment in our microarray analysis (Fold-Change = − 6.66; adjusted *p*-value 0.0012). Furthermore, the TGF-β signaling resulted also significantly modulated by Crenigacestat in the pathway analysis previously reported (Fig. 1). As shown in Supplementary Fig. 1, Crenigacestat inhibited TGF-β via the canonical pathway. To confirm our bioinformatic analysis, we investigated the TGF-β pathway in PDX models treated with Crenigacestat or vehicle. TGFB1 gene expression was significantly (*p* < 0.05) downregulated by Crenigacestat treatment compared to vehicle (Fig. 4A). Consistently, TGF-β and phospho-Smad-2 protein expression were significantly (*p* < 0.001) reduced following drug treatment (Fig. 4B e C).

**Fig. 5:**
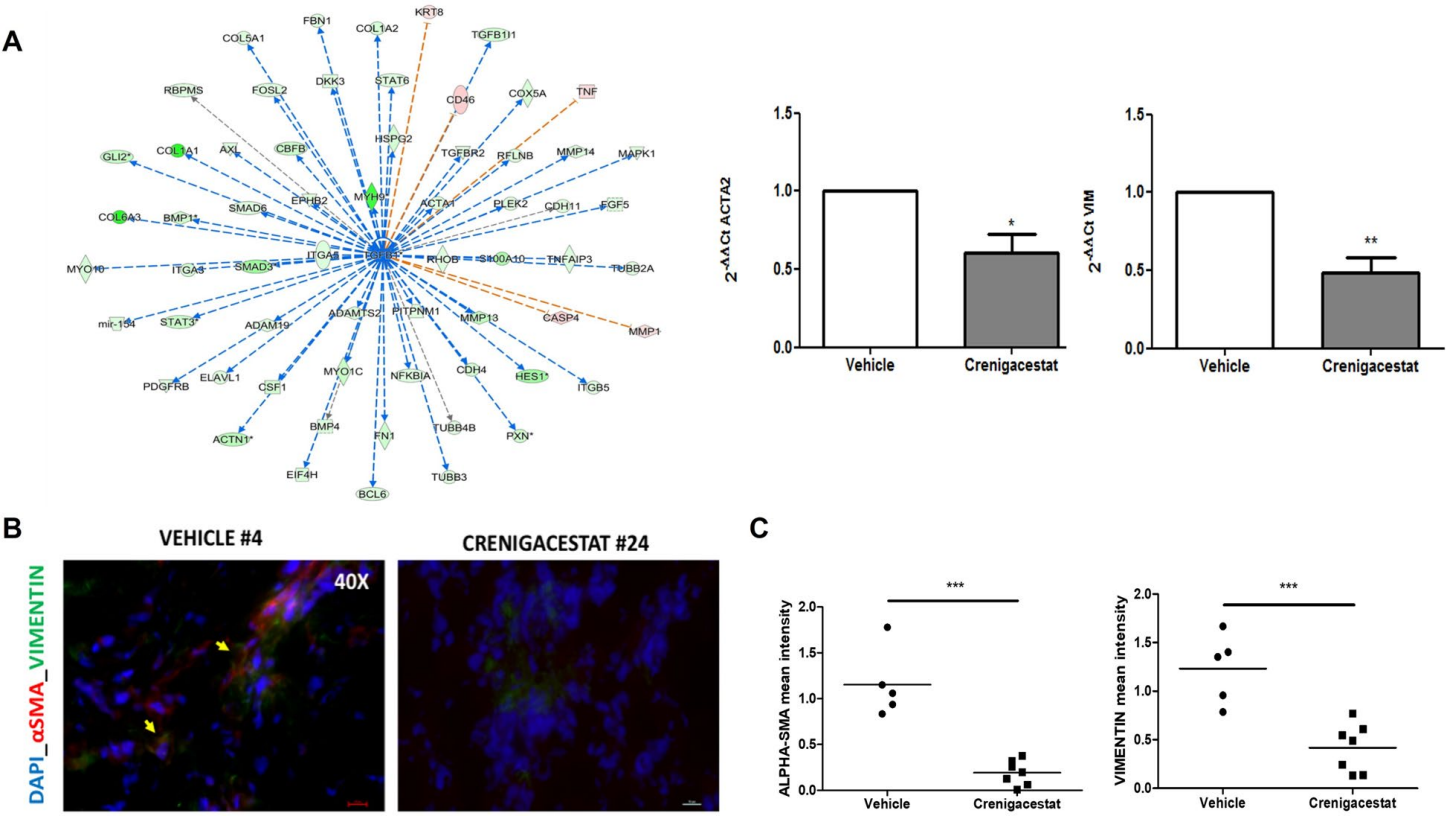
Crenigacestat reduces iCCA hCAFs activation in PDX tissues. Panel **A**. TGFB1 as predicted upstream regulator and its target molecules by Ingenuity Pathway Analysis (IPA) in treated vs. untreated PDX tissues. Genes in red denote upregulation and downregulation in response to the treatment in green. Lines in orange denote predicted activation; lines in blue predict inhibition. Panel **B**. Crenigacestat downregulates α-SMA expression in PDX-treated mice, as detected by immunofluorescence staining. Vimentin (green) and α-SMA (red) in overlapping stains (yellow) co-immunolocalize in PDX tissues. The scale bar represents 50 μm. Panel **C**. The staining quantification was calculated as the mean intensity fluorescence of three images/tissue from PDX mice treated with Crenigacestat compared to the vehicle. ***p < 0.0001. Magnifications: 40X

**Fig. 6:**
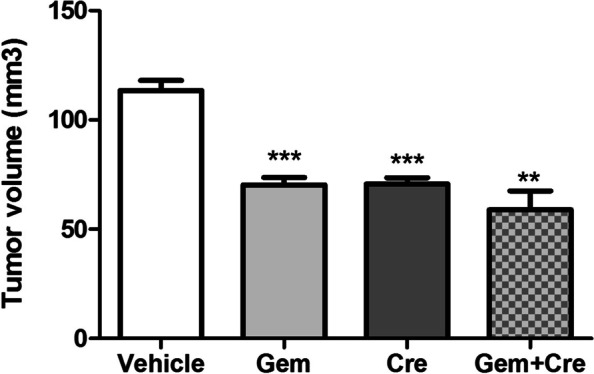
Crenigacestat enhances the chemosensitivity of iCCA to Gemcitabine in the PDX model**.** At the end of treatment, the tumor volume of the masses was significantly reduced in all treated-mice compared to the vehicle. The combination of Crenigacestat with Gemcitabine improves the efficacy of treatment in iCCA. **p < 0.001, ***p < 0.0001 calculated with Student’s t-test

To better understand the involvement of the TGF-β pathway while inhibiting Notch signaling, we in silico analyzed the upstream regulator of differentially expressed genes modulated by Crenigacestat in PDX tissues. In this analysis, one of the most significant upstream regulators predicted to be associated with differentially expressed genes was TGFB1 (z-score − 4.751, *p*-value 3.36E-16). The predicted relationship between TGF-β1 and HES1 was particularly exciting (Fig. 5A). The connection between TGF-β1 and NOTCH pathways was further confirmed in the network generated with NOTCH1 as the upstream regulator (Supplementary Fig. 2). Furthermore, the previously reported upstream regulator analysis predicted that TGFB1 modulates ACTA1 expression (Fig. 5A). To validate this finding, we investigated the mRNA expression of ACTA1 isoform, ACTA2, and Vimentin (VIM) in the same PDX tissues previously studied. As reported in Fig. 5A, both genes were significantly downregulated (*p* < 0.01 and *p* < 0.001, respectively). To further confirm our results at protein levels, we immunolocalized α-smooth muscle actin (α-SMA) and Vimentin by immunofluorescence staining. As reported in Fig. 5B, α-SMA and Vimentin were expressed in the tissue surrounding the tumor, and their colocalization suggests the presence of human CAFs. The staining intensity quantification of both proteins was significantly (*p* < 0.0001) reduced by Crenigacestat treatment (Fig. 5C). These results suggest that inhibition of the Notch pathway also downregulates TGF-β and α-SMA expression. To assess the functional role of suppressing the Notch pathway for iCCA treatment and as a chemosensitizer, we treated the PDX model with Crenigacestat and Gemcitabine, used alone or in combination. As reported in Fig. 6, Crenigacestat chemosensitized iCCA to Gemcitabine in the PDX model.

**Fig. 7:**
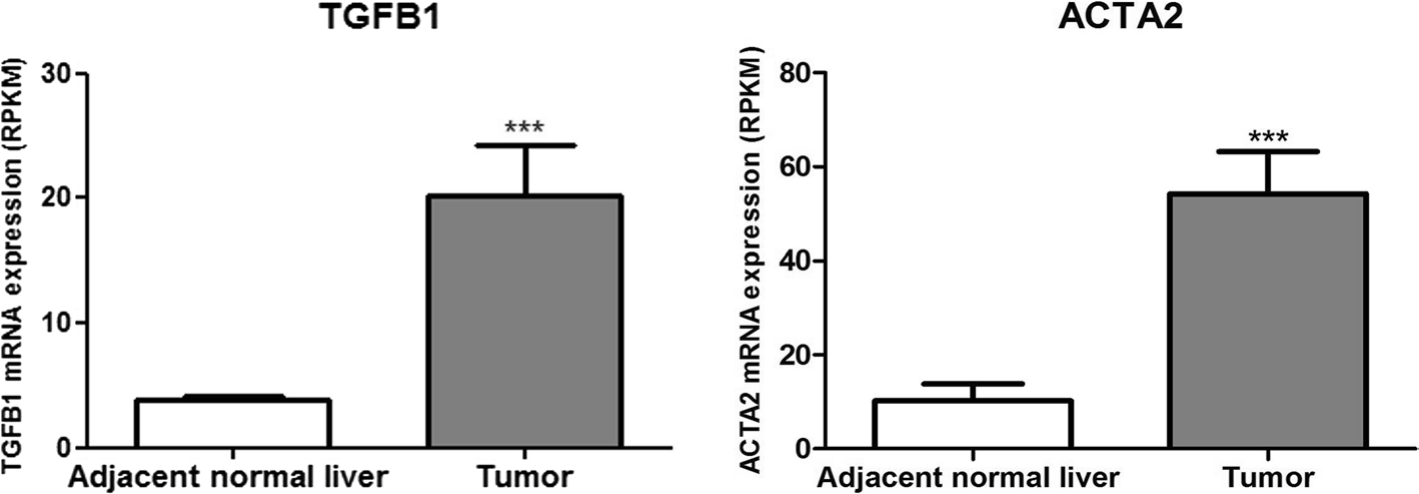
Analysis of TGFB1 and ACTA2 mRNA expression in tumoral and matching peritumoral tissues of 31 iCCA patients from the GEO database (GSE107943). Mean expression data in RPKM (Reads Per Kilobase Million). ***p < 0.0001 calculated with Student’s t-test

To explore the hypothesis that both TGF-β and α-SMA may take a role in patients, we investigated their mRNA levels in tumoral and peritumoral tissues of 31 iCCA patients from the GEO database (GSE107943) [37]. As reported in Fig. 7, both TGFB1 and ACTA2 (α-SMA) were significantly upregulated (*p* < 0.0001) in the tumoral compared to pair peritumoral tissues.

In conclusion, Crenigacestat inhibits TGF-β1 and deactivates CAFs in the microenvironment tissue surrounding iCCA in a PDX model.

### Crenigacestat inhibits notch and TGF-β1 signaling in hCAFs

**Fig. 8:**
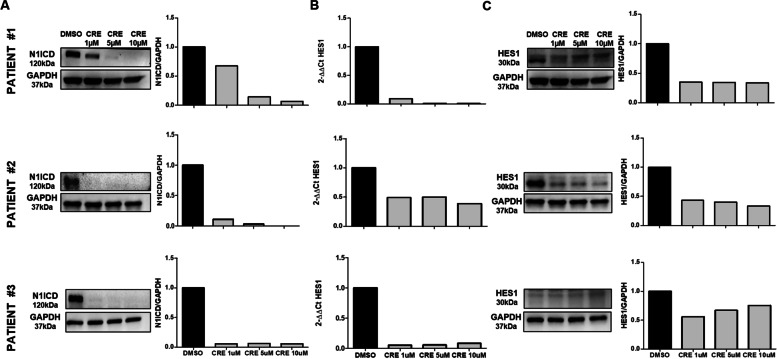
Crenigacestat inhibits the NOTCH1 pathway in iCCA hCAFs. Panel **A.** Crenigacestat inhibits the intracellular domain of Notch1 (NICD1) in a dose-dependent manner. Panel **B** and **C.** HES1, at both mRNA and protein expression, is downregulated in iCCA hCAFs at all the concentrations used of Crenigacestat. *p < 0.05, **p < 0.01, ***p < 0.001 calculated with Student’s t-test

**Fig. 9:**
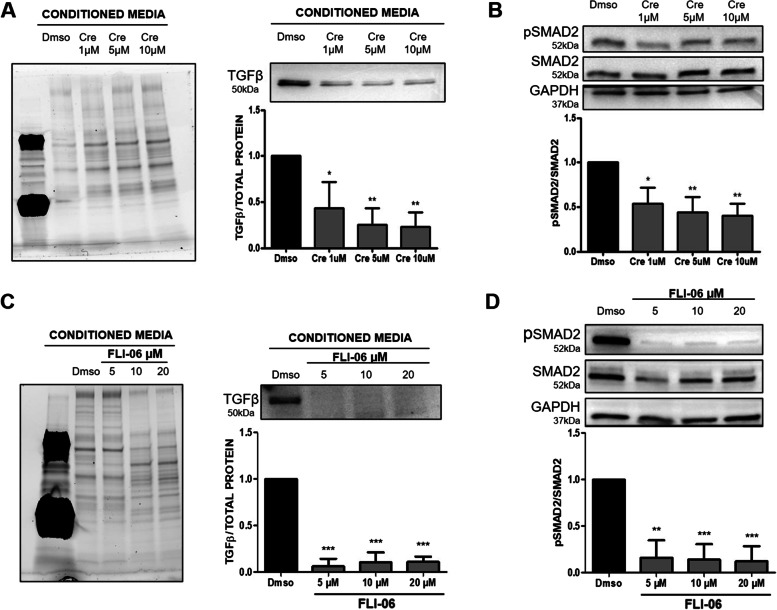
Notch inhibitors downregulate TGFβ pathway in iCCA hCAFs. Panel **A** and **C.** Crenigacestat and FLI-06 downregulate TGFβ secretion in iCCA hCAFs conditioned media. Panel **B** and **D**. pSmad2 was significantly inhibited by both Notch inhibitors; in particular, Crenigacestat induces a dose-dependent inhibition. *p < 0.05, **p < 0.01, ***p < 0.001 calculated with Student’s t-test

To get better insight into the molecular mechanism, we isolated and characterized, as previously described [35], human primary CAFs from patients with iCCA, and challenged them with Crenigacestat. Drug treatment significantly (*p* < 0,0001) blocked the intracellular domain of Notch1 (NICD1) in a dose-dependent manner (Fig. 8A). Hairy and enhancer of split-1 (HES1) gene, a relevant gene for Notch signaling, was also downregulated at mRNA and protein levels at all the concentrations used (*p* < 0.0001 and *p* < 0.05, respectively) (Fig. 8B and C respectively). This finding suggests that CAFs are also a target of Crenigacestat. In the same CAF preparations in serial experiments, drug treatment significantly reduced TGF-β protein expression (*p* < 0.05) at all the concentrations used (Fig. 9A). Consistently, pSmad2 was also significantly (*p* < 0.05) inhibited in a dose-dependent manner (Fig. 9B), suggesting that Crenigacestat inhibits TGF-β signaling in CAFs. To further confirm previously described results, CAFs were challenged under the same experimental conditions with FLI-06, a Notch inhibitor that disrupts the Golgi apparatus inhibiting its secretion from the endoplasmic reticulum, an earlier stage with respect to the γ-secretase activation [38, 39]. Consistently, TGF-β1 and p-Smad-2 activation levels were statistically (*p* < 0.001) reduced (Fig. 9C and D, respectively).

In conclusion, Crenigacestat inhibits Notch and TGF-β1 signaling cascades in hCAFs freshly isolated from patients with iCCA.

### Crenigacestat inhibits the secretion of ECM components by hCAFs

**Fig. 10:**
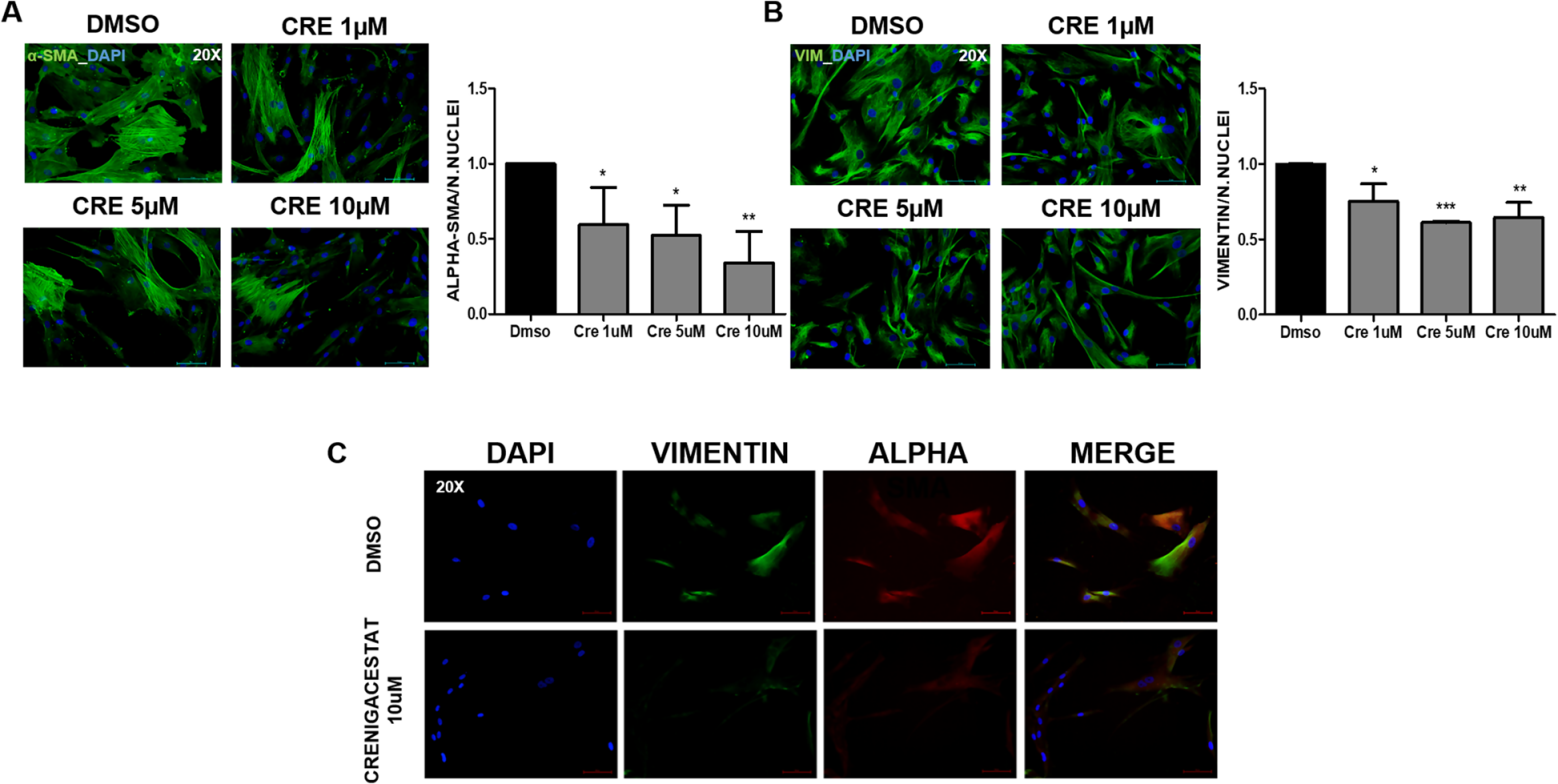
Crenigacestat inactivates iCCA hCAFs. Crenigacestat downregulates α-SMA (green signal, Panel **A**) and Vimentin (green signal, Panel **B**) expression in treated iCCA hCAFs, as detected by immunofluorescence staining. For both protein markers, staining quantification was calculated on nuclei number as the mean of five images per cell slide treated with increasing Crenigacestat concentrations compared to untreated. The scale bar represents 10 μm, magnification 20x. Panel **C**. Vimentin (green) and α-SMA (red) in overlapping stains (yellow) were co-immunolocalized in the same primary cell cultures. The scale bar represents 50 μm, magnification 20x.*p < 0.05, **p < 0.001, ***p < 0.0001 calculated with Student’s t-test

To investigate Crenigacestat effectiveness on hCAFs, we evaluated α-SMA and Vimentin expression in hCAFs. As reported in Fig. 10, Crenigacestat significantly downregulated α-SMA and Vimentin expression (*p* < 0.05) in hCAFs at all concentrations used. In addition, in the control group, α-SMA and Vimentin colocalized, as previously described in PDX tissues (Fig. 10C), whereas Crenigacestat treatment completely abrogates such colocalization (Fig. 10C). These results suggest that drug treatment deactivates CAFs.

**Fig. 11:**
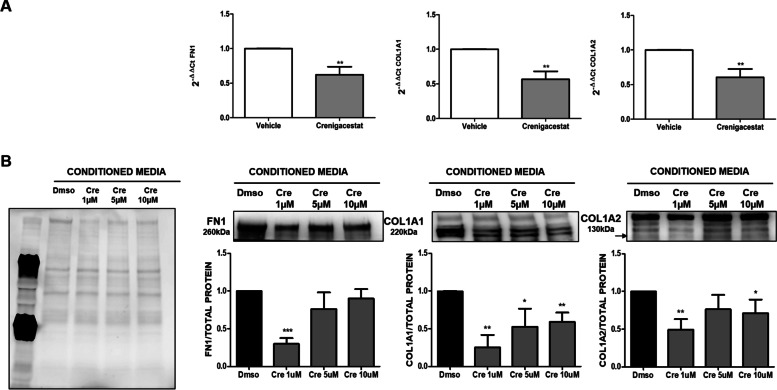
Crenigacestat reduces ECM component in iCCA hCAFs. Panel **A**. Real-time PCR for FN1, COL1A1, and COL1A2 on PDX mice reveals a reduction of ECM genes in PDX treated mice. Panel **B**. Crenigacestat inhibits the ECM protein deposition in iCCA hCAFs, in particular, Crenigacestat effect is more evident on Fibronectin at the lowest dose and COL1A1 at all concentrations. *p < 0.05, **p < 0.01, ***p < 0.001 calculated with Student’s t-test

Finally, to recapitulate the effectiveness of Crenigacestat in PDX models, we evaluated the effect of Crenigacestat treatment on ECM genes. Notably, the expression of FN1, COL1A1, and COL1A2 was significantly reduced after Crenigacestat treatment (*p* < 0.01, Fig. 11A). Moreover, we investigated the secretion of ECM components such as Fibronectin, Collagen 1A1, and Collagen 1A2 by hCAFs isolated from three different patients following drug treatment. As reported in Fig. 11B, Crenigacestat inhibited the secretion of all ECM proteins, although the effect was more evident regarding Fibronectin at 1 μM and COL1A1 at all drug concentrations, consistent with PDX transcriptomic data reported in Figs. 5A and 11A.

In conclusion, according to our bioinformatic analysis and experimental results, Crenigacestat inhibits Notch signaling and TGF-β1 associated with dephosphorylation of SMAD2/3. This induces the deactivation of CAFs, with the consequent reduction in the secretion of ECM components (COL1A1, COL1A2, and FN1) and liver fibrosis.

## Discussion

iCCA is a highly locally invasive malignant tumor causing a poor patient prognosis. Drug-based therapeutic choices are very limited and almost ineffective against this disease [40, 41]. Cancer cells can respond to drug treatment by activating complex mechanisms of chemoresistance (MOC). These MOC allow tumor cells to circumvent the potentially harmful effects of chemotherapy [42]. Recent findings highlight the crucial role of the inflammatory milieu, because of EGFR-RAS-MAPK axis activation and pro-carcinogenic cytokine IL6 production, in iCCA progression [43]. Molecular mechanisms underlying resistance to treatment are still unknown; nevertheless, iCCA is characterized histologically by a strong desmoplastic reaction [9, 44, 45]. The surrounding microenvironment has been reported to facilitate tumor progression, mainly because of the production and deposition of ECM proteins [46] and the crosstalk with cancer cells [46]. For instance, in HCC, ECM proteins, such as Ln-332, provide resistance to Sorafenib and take a crucial role in creating the ideal cancer stem cell niche [47]. CAFs are therefore implicated in cancer progression; indeed, the expression of α-SMA, a marker commonly used to assess active myofibroblasts, is more expressed in the tumoral than paired nontumoral tissues of patients with different malignancies, including HCC [48] In 31 iCCA patients, we also report that α-SMA expression is more pronounced in the tumor than in peritumoral tissues, consistent with previous literature. In the same patients, we also showed that TGF-β is more expressed in tumoral than in peritumoral tissues, being TGF-β the most potent activator of fibroblasts toward CAFs [49–51]. TGF-β is also overexpressed in the iCCA stroma, and its levels correlate with iCCA patients’ overall survival [52].

The Notch pathway drives the development and morphogenesis of bile ducts, while its overexpression is responsible for CCA onset in experimental mouse models [30]. We have recently shown that the Notch signaling inhibitor Crenigacestat reduces tumor growth of iCCA expressing high levels of CD90 in experimental mouse models [33]. In the same experimental condition, herein, we demonstrate for the first time that inhibiting Notch pathway also affects tumoral surrounding liver fibrosis. In particular, we show that Crenigacestat modulates TGF-β expression. The crosstalk between Notch and TGF-β signaling pathways occurs at multiple levels and in various cellular contexts. Indeed, many processes that are regulated by Notch signaling are also controlled by TGF-β family ligands [53–56]. Several studies have demonstrated that Notch and TGF-β are involved in forming bile ducts and in the differentiation of cholangiocytes [57, 58] since some mediators of these two pathways were increased [59]. In other cases, Notch signaling antagonizes growth arrest and transcription induced by TGF-β [60, 61]. These opposite results highlighted the complexity of these two networks and the influence of cell-specific cofactors. In the present work, we demonstrate, for the first time, that in iCCA stroma, the inhibition of Notch signaling by Crenigacestat leads to TGF-β decrease via Smad2 phosphorylation, suggesting a positive correlation between the two pathways. These data were further confirmed also in CAFs isolated from different patients with iCCA. Crenigacestat inhibited TGF-β secretion and pSmad2, resulting in the inactivation of CAFs, documented by reduction of α-SMA and consequent decrease of ECM proteins, such as FN, COL1A1, and COL1A2, secretion. Recently, it has been reported TGF-β pathway also interacts with Notch signaling in cholangiocarcinogenesis [54]. The functional crosstalk between Notch and TGF-β signaling occurs via direct interaction with NICD1 that recruits the TGF-β downstream signaling mediators, Smads [54]. Here, we found that in iCCA hCAFs, Crenigacestat inhibits Notch and TGF-β1 signaling. Specifically, Crenigacestat treatment blocked the cleavage of NICD1 and the downstream transcription factor HES1. Accordingly, the canonical pathway of TGF- β was affected by treatment since TGF-β and Smad2 expression was reduced.

In this study, we demonstrate that inhibiting Notch signaling restores homeostasis in the ecosystem surrounding iCCA, exposing tumoral cells to drug effectiveness, and this is a new therapeutic target. For instance, Crenigacestat increases cisplatin’s effects in experimental gastric cancer models and sensitizes otherwise resistant cells [62]. Furthermore, in osteosarcoma patient-derived primary tissues, the inhibition of the Notch pathway downregulates stemness-related gene expression, such as CD133, likely by remodeling the surrounding tumor microenvironment [63].

## Conclusions

In conclusion, for the first time, we demonstrate that the liver fibrotic component of the iCCA microenvironment is controlled by the Notch signaling through the TGF-β canonical pathway, although we cannot rule out the possibility of a direct effect on hCAFs. This envisages a new potential strategy to fight tumor progression whereby a Notch inhibitor could be associated with other drugs to improve effectiveness.

## Supplementary Information

**Additional file 1.**

## Data Availability

All data generated or analyzed during this study are included in this manuscript [and its supplementary information files].

## Abbreviations

iCCA: Intrahepatic cholangiocarcinoma
ECM: Extracellular matrix components
TGF-β1: Transforming growth factor
α-SMA: α-smooth muscle actin
CAFs: Cancer-associated fibroblasts
COL 1A1: Collagen type 1A1
COL 1A2: Collagen type 1A2
FN: Fibronectin
HSCs: Hepatic Stellate Cells
EMT: Epithelial-mesenchymal transition
NICD: NOTCH Intracellular Domain
HES-1: Helix-loop-helix transcription factor
GSI: γ-secretase inhibitor
PDX: Patient-Derived Xenograft
