# Supplementary material for: Crenigacestat blocking notch pathway reduces liver fibrosis in the surrounding ecosystem of intrahepatic CCA viaTGF-β inhibition
Source: J Exp Clin Cancer Res. 2022 Nov 28;41:331. doi: 10.1186/s13046-022-02536-6 (PMC9703776; doi:10.1186/s13046-022-02536-6)
Supplement: Supplementary file 1 — Additional file 1. [file 13046_2022_2536_MOESM1_ESM.docx]

**Article Type: Original Article**

**Blocking Notch pathway reduces liver fibrosis in the surrounding ecosystem of intrahepatic CCA viaTGF-β inhibition**

**Serena Mancarella^1✝^, Isabella Gigante^1✝^, Grazia Serino^1^, Elena Pizzuto^1^, Francesco Dituri^1^, Maria F. Valentini^2^, Raffaele Armentano^1^, Diego Calvisi^3^, Gianluigi Giannelli^1^**

*^1^National Institute of Gastroenterology "S. de Bellis", Research Hospital, Castellana Grotte, Italy*

*^2^Department of Emergency and Organ Transplant, University of Bari Medical School, Bari, Italy*

*^3^Institute of Pathology, University of Regensburg, Regensburg 93053, Germany*

***Email:***

*serena.mancarella@irccsdebellis.it,*

*isabella.gigante@irccsdebellis.it,*

*grazia.serino@irccsdebellis.it,*

*elena.pizzuto@irccsdebellis.it,*

*francesco.dituri@irccsdebellis.it,*

*mariapiavalentini@gmail.com,*

*raffaele.armentano@irccsdebellis.it,*

*diego.calvisi@ukr.de,*

*gianluigi.giannelli@irccsdebellis.it*

***✝*** *Serena Mancarella and Isabella Gigante contributed equally to this work*

***Correspondence:*** Prof. Gianluigi Giannelli, National Institute of Gastroenterology “S. De Bellis” Research Hospital, Via Turi 27 - 70013 Castellana Grotte (BA), Italy, Phone +390804994175, [gianluigi.giannelli@irccsdebellis.it](mailto:gianluigi.giannelli@irccsdebellis.it) (G.G.)

**Figure S1**


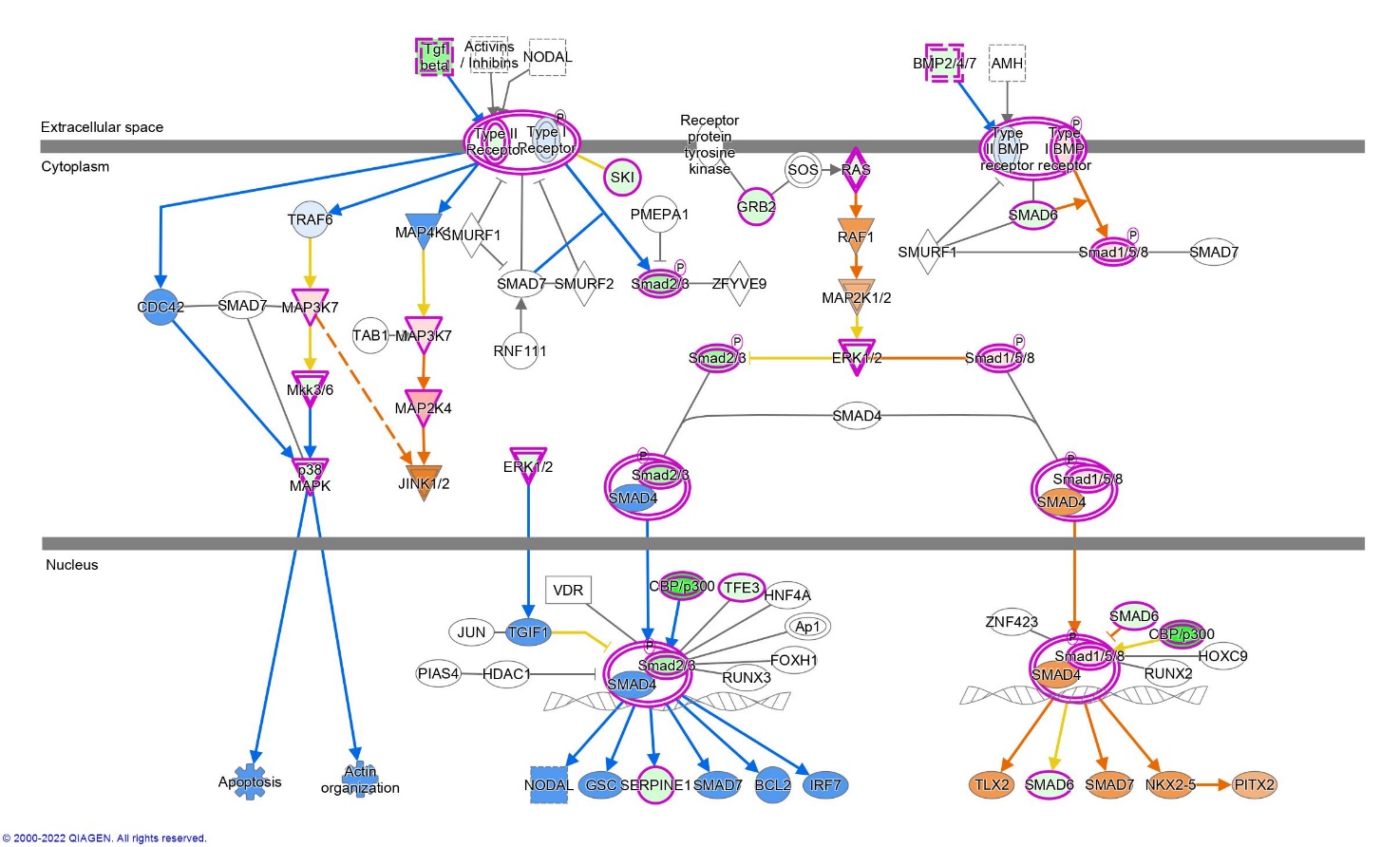


**Figure S1. Bioinformatic analysis reveals the involvement of TGFβ signaling in PDX tissues treated with Crenigacestat.**

**Figure S2**


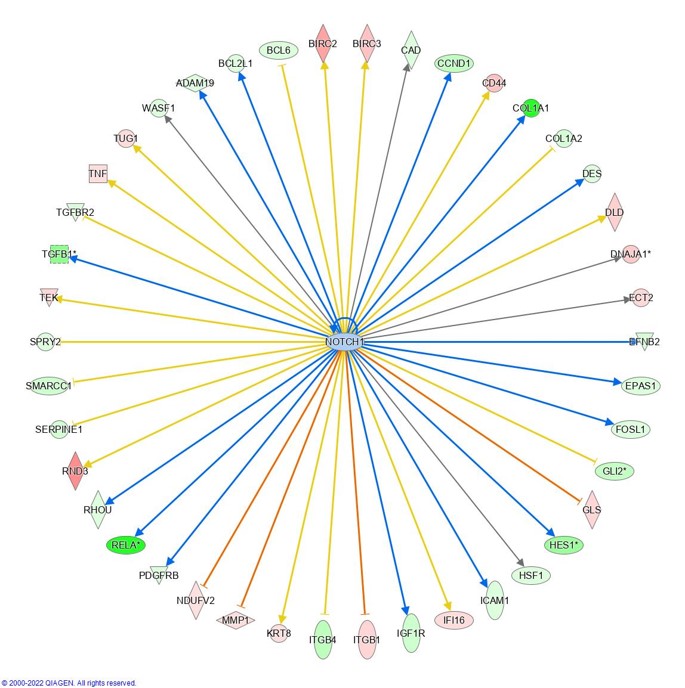


**Figure S2.** Notch1 as predicted upstream regulator of Crenigacestat treatment and its target molecules by Ingenuity Pathway Analysis (IPA). Genes in red denote up-regulation and in green down-regulation in response to the treatment. Lines in orange denote predicted activation; lines in blue predicted inhibition.
